# Quality of life, wellbeing, recovery, and progress for older forensic mental health patients: a qualitative investigation based on the perspectives of patients and staff

**DOI:** 10.1080/17482631.2023.2202978

**Published:** 2023-04-20

**Authors:** Kate Walker, Jen Yates, Tom Dening, Birgit Völlm, Jack Tomlin, Chris Griffiths

**Affiliations:** a Research and Innovation, Northampton Healthcare NHS Foundation Trust, Northampton, UK; b Institute of Mental Health, University of Nottingham, Nottingham, UK; c Klinik und Poliklinik für Forensische Psychiatrie, University Hospital of Rostock, Rostock, Germany; d School of Law and Criminology, University of Greenwich, London, UK

**Keywords:** Forensic mental health, older patients, quality of life, recovery, service provision

## Abstract

**Purpose:**

There is a lack of research informing service requirements for older (aged≥55 years) forensic mental health patients. The aim of this research was to increase knowledge about older forensic mental health patients’ quality of life, wellbeing, recovery, and progress, in order to make recommendations of how to facilitate and enhance these factors.

**Methods:**

In-depth interviews with patients (*N* = 37) and staff (*N* = 48) were undertaken; data were analysed using thematic analysis.

**Results:**

Environmental (e.g., physical, structural and facilities), relational (staff, family and friends) and individual (characteristics, feelings, behaviours) factors were identified as enablers and/or obstacles to wellbeing, recovery, progress and quality of life.

**Conclusions:**

The physical and psychological environment of services needs to be adapted to meet the needs of patients. Therapeutic relationships with staff should be encouraged and a person-centred and individual recovery approach adopted. Prosocial relationships with peers, friends and family need to be fostered to enable positive recovery outcomes. Older patients should be empowered to develop a sense of autonomy to enable quality of life, wellbeing, and recovery, and progress.

## Introduction

Changes in demographic trends, including longer life expectancy (World Health Organization, (WHO (2017)), increased life prison sentences, indeterminate prison sentences, mandatory sentencing (Senior et al., 2013) and increases in convictions in later life (such as for historical offences) (House of Commons Justice Committee, 2013), mean there is an increasing number of older patients accessing forensic mental health services. Older forensic mental health patients comprise three distinct subgroups: those who are old when they commit their first offence, those whose offending started when they were much younger and who have continued to reoffend throughout their lives (recidivists), and those who offended young and have remained detained for decades (Ulmer & Steffensmeier, 2014). In the UK and other European countries, around 20% of patients in secure settings are over 50 years old (DiLorito et al., 2018, 2019). Forensic mental health services represent an intersection between health and justice systems, which creates a complex care environment. Currently in the UK there is no specific separate provision for older forensic patients in forensic mental health services (DiLorito et al., 2018, 2019).

Older forensic mental health patients’ needs are complex, and this is likely to impact on their quality of life (QoL). Older forensic mental health patients are often diagnosed with severe mental disorders such as schizophrenia, personality disorder, bipolar disorder, or major depressive disorder (Huband et al., 2018), have increased risk of co-morbid conditions (DiLorito et al., 2019) and chronic physical illnesses, e.g., cardiovascular disease, diabetes, chronic obstructive pulmonary disease (COPD) (Ivbijaro et al., 2008; Lightbody et al., 2010) and cognitive difficulties, e.g., mild cognitive impairment (MCI) and dementia (Ribe et al., 2015). They are likely to have been on antipsychotic medication long-term and are therefore at increased risk of side effects such as weight gain, diabetes and dyslipidaemia (DeJongh, 2021). Unhealthy lifestyles that include smoking, problematic alcohol use, high calorie and poor nutritional diets, and physical inactivity are also features of these patients’ lives (Pedersen et al., 2020). They also experience natural changes that are associated with ageing such as reduced/poor mobility, frailty, and deteriorating eyesight and hearing (Natarajan & Mulvana, 2017).

NHS England commission approximately 7700 inpatients beds in secure mental health services; approximately 800 in high secure, 3200 in medium security and 3700 in low security (NHS England, 2019). In-patient forensic mental health services are designed to offer a therapeutic environment, that provides mental and physical health care and treatment, and interventions to reduce risk and address criminogenic needs (Tomlin & Jordan, 2021) to enable individuals to progress (conceptualized here as a reduction in restrictiveness and more independent living). Community-based forensic mental health services are also provided (Natarajan & Mulvana, 2017); these services offer specialist psychological and psychiatric support, to assess, manage and treat individuals (who as a consequence of mental illness have offended) in the community (generally their own home settings). The focus is on offering the least restrictive care as possible, providing an alternative to secure inpatient settings. Some forensic settings are highly restrictive: patients’ freedom and independence are curbed; autonomy, choice and decision making are restricted; visits from family and friends must be planned, agreed and authorized; and routines such as mealtimes and recreational times are fixed (Markham, 2018; Tomlin et al., 2018). Patients find themselves in environments where social connections are complex: with staff and professionals having a dual role of care and custody (Joyes et al., 2021), where they are surrounded by other patients (peers) due to circumstances and not necessarily by choice, and they are without their external community-based family and friends (with whom contact is restricted).

Recovery is a process of living with and through a mental disorder, and defined as inherently subjective (Tomlin & Jordan, 2021). In the current research recovery is defined as the personal journey of forensic mental health patients as they pursue their own, unique, life goals with or without continuing symptoms. A recovery-oriented model of care is well-established as a preferred treatment framework in mental health provision, with forensic mental health services now embracing recovery principles (McKenna et al., 2014). Here the focus is on personal recovery, which argues against just treating symptoms, placing a focus on developing resilience; this recovery is a process that is personal, unique and a way of living a satisfying life, within the limitations caused by mental disorder (Jacob, 2015). Leamy et al. (2011) developed a conceptual framework of personal recovery in mental illness, with the acronym of CHIME which represents five recovery processes: connectedness; hope and optimism about the future; identity; meaning in life and empowerment. Specifically, in forensic mental health Senneseth et al. (2022), identified an additional recovery process which was related feeling safe and being secure (safety and security), providing the CHIME-Secure framework (CHIME-S). This offers a framework for understanding personal recovery in forensic populations and identifies areas that are likely to be of relevance for the older forensic mental health patients, who are the focus of the current research.

It has been suggested that patients evaluate QoL in forensic hospitals quite differently, some finding that being detained and restricted has an exceptionally negative association with QoL, whereas others say it can be improved as the structured environment is beneficial and protective (Büsselmann et al., 2021). QoL has been found to differ in the context of forensic mental health institutions, based on conditions within the establishments (e.g., quality of accommodation, therapeutic options), and individual characteristics such as age, and type and severity of mental health (Büsselmann et al., 2021). QoL is difficult to define and measure, it is a subjective experience and can mean different things to different people. In the current research QoL was conceptualized based on previous research which suggests that key domains for QoL for older adults are: “autonomy, role and activity, health perception, relationships, attitude and adaptation, emotional comfort, spirituality, home and neighbourhood, and financial security” (Van Leeuwen et al., 2019, p. 1). It also took into consideration that for people with mental health conditions, QoL involves domains including: wellbeing; control; autonomy and choice; self-perception; belonging; activities; and hope (Connell et al., 2012). Patients who are long-stayers within forensic settings, (for example, inpatients for>5 years or >10 years in medium and high security respectively; Hare Duke et al., 2018) have particular requirements for the maintenance of QoL, including mental and physical health treatment, provision of daytime activities, and interventions to improve social skills and self-esteem (Glorney et al., 2010). The extent to which QoL in forensic mental health services for older patients matches these definitions and requirements is unknown. In this article the aim is to qualitatively explore understand, and increase knowledge on what contributes or hinders QoL, wellbeing, recovery and the thrive to progress in forensic psychiatric care settings (inpatients and community), based on patients’ and staff’s narratives and perspectives. This addresses our research question, namely, what enables (what works) older forensic mental health patients to progress in terms of improvement in quality of life, wellbeing, and recovery and what are the barriers (what doesn’t work) and facilitators associated with this process?

## Method

### Design

This research took a qualitative design, where semi-structured interviews were conducted with a purposive sample of healthcare professionals working in NHS inpatient or community forensic health services, and older forensic mental health inpatients and those under NHS care living in the community.

### Participants

Data were collected (from March 2020 to August 2021) through in-depth interviews with 37 older (aged≥55 years) forensic mental health patients (in-patients and in the community) and 48 staff and professionals working with this patient group, from eight NHS Trusts in England. The authors estimated sample size based on their discussions on information power. Numbers we based on the appraisal model advocated by Malterud et al. (2015) whereby the following five dimensions were considered: (i) study aim, (ii) sample specificity, (iii) use of established theory, (iv) quality of dialogue, and (v) analysis strategy. Purposive sampling was used to recruit patients to represent different settings (high, medium, low secure, community) and different professions of staff. Table I presents demographic information for the sample.

**Table I.** Sample demographics.

PATIENTS (*N* = 37)		STAFF (*N* = 48)	
**Age**	*M = 59.8 SD = 3.9*	**Professions**	
**Gender**		Psychiatrists (PMD)	*n =* 7 (15%)
Male	*n* = 34(92%)	Psychologists (Psy)	*n =* 7 (15%)
Female	*n* = 3(8%)	Occupational Therapists (OT)	*n =* 8 (17%)
**Ethnicity**		Physiotherapist (Phy)	*n = 1* (2%)
White	*n* = 30(81%)	Social Workers (SW)	*n = 5* (10%)
Black, African, Caribbean, Black British	*n* = 6(16%)	Community Registered Mental .Nurses (C/RMN)	*n = 5 (10%)*
Mixed or multiple ethnic group	*n* = 1(3%)	Inpatients Registered Mental .Nurses.(RMN)	*n = 12* (25%)
		Non-Clinical Staff (N/Clin)	*n =* 3 (6%)
**Setting**		**Setting**	
High secure	*n* = 10(27%)	High secure (HS)	*n =* 8 (17%)
Medium secure	*n* = 9(24%)	Medium secure (MS)	*n =* 19 (40%)
Low secure	*n* = 8(22%)	Low secure (LS)	*n =* 13 (27%)
Community	*n* = 10(27%)	Community (C)	*n =* 8 (17%)
**Mental health diagnosis ordered by ICD-10 categories1**			
Organic, including symptomatic, mental disorders	*n* = 1(3%)		
Mental and behavioural disorders due to psychoactive substance use	*n* = 5(14%)		
Schizophrenia, schizotypal and delusional disorders	*n* = 22(60%)		
Mood [affective] disorders	*n* = 6(16%)		
Neurotic, stress-related and somatoform disorders	*n* = 3(8%)		
Personality disorders (Any)	*n* = 15(41%)		
*Dissocial*	*n* = 5(14%)		
*Dependent*	*n* = 3(8%)		
*Avoidant (anxious)*	*n* = 5(14%)		
*Emotionally Unstable*	*n* = 4(11%)		
*Paranoid*	*n* = 4(11%)		
*Schizoid*	*n* = 2(5%)		

^a^Observations greater than 37 and percentages greater than 100 as most patients had multiple diagnoses. *N* = 37.

### Procedure

Ethical approval was granted by the Health Research Authority (HRA) (IRAS project ID: 258016; research ethics committee [REC] reference: 19/EM/0350). Recruitment of participants was through the support of National Institute for Health and Care Research’s (NIHR) Clinical Research Network (CRN), who provided Principal Investigators (PIs) responsible for local site application of recruitment protocol, ensuring potential participants who met inclusion criteria were offered the opportunity for participation. Participants were invited to participate by PIs at each site and were given a participant information sheet to read. They were given time to consider participation before they were asked to give informed consent. It was explained to all participant about the voluntary nature of participation and that they could withdraw at any point without providing an explanation and—for the patients -without their care and treatment or legal rights being affected. All participants provided informed written or recorded verbal consent prior to the interview. Participants attended a one-to-one semi-structured interview, either face-to-face (staff *n* = 2, patients *n* = 9), video call (staff *n* = 46, patients *n* = 27) or telephone call (patients *n* = 1). One member of the research team undertook all the interviews. Face to face and video interviewing methods yielded rich data. The one telephone interview undertaken was deemed more difficult for facilitating the flow of the discussion as this method does not allow for the use non-verbal cues (nodding, smiling, eye-contact); however, the interview still yielded rich and detailed data. The semi-structured interview questions were developed based on existing literature in the field, input from the research team, a Lived Experience Advisory Panel (LEAP; all service or ex-service users/patients), and an expert professional advisory panel. The interviews were piloted with LEAP members to assess if the questions were relevant, appropriate, easy to understand and able to elicit descriptive responses. The interview questions were worded to gather information on quality of life, health and wellbeing, interventions and activities, age, and progress and prompts and adjunctive questions were used to gain more information as needed. Example questions for patients included: In your current situation what facilitates/enables (has helped) you to have good quality of life?; What maintains your mental health/physical health/wellbeing?; What do you think is needed specifically for older patients?; How would you describe what progress looks like for you? For the staff example questions included: How would you know that an older patient did not have good quality of life?; Can you describe if there is anything specifically tailored for supporting older patients’ mental health/physical health/wellbeing?; Do you perceive older patients as different or the same as younger patients and why?; How do you know if older patients are not progressing? (Interview schedules are available from authors on request). Staff interview length ranged from 36 minutes to 106 minutes and from 28 minutes to 80 minutes for the patients. All interviews were transcribed verbatim, anonymized, and uploaded to NVivo (V.20) (QSR international, 2020) for analysis.

### Ontological and epistemological assumptions

The ontological and epistemological assumptions were critical realism (retaining a concept of reality and truth, while acknowledging human practices will shape how we experience this) and contextualism (where knowledge and those who created it, are contextually embedded, perspectival and partial, and where multiple accounts of reality are viable; Braun & Clarke, 2021). Critical realism is concerned with the nature of causation, structure, agency and relations, that help identify causal mechanism during social events, activities, or phenomena (Danermark et al., 2001); in the current research what produces the mechanisms that underpin quality of life, wellbeing, recovery, and progress for older forensic mental health patients was sought. Critical realism is suited for thoughtful in-depth research that aims to understand why things are as they are (Easton, 2010).

### Data analysis

Thematic analysis as described by Braun and Clarke (2021) was utilized to analyse the data, and the process was data driven and inductive and focused on the semantic level to capture explicitly expressed meaning (Braun & Clarke, 2021). An inductive approach was chosen as the research was exploratory in nature, and the key aim was to examine and understand experiences, perspective, meanings, and participants’ articulated narratives that form the starting point for coding and theme development. Thematic network analysis (Attride Stirling, 2001), a type of thematic analysis, was used to identify basic, organizing, and global themes.

Staff and patient data were analysed and interpreted the same way. After familiarization with the data by reading and re-reading the interviews, initial coding was undertaken to describe the data in detail, followed by secondary axial coding to start forming broader codes based on terms and concepts found in the data. This process was done by two members of the research team, and for a selection of transcripts, the LEAP members also undertook initial coding. The initial and axial codes were then developed into themes, which were then reduced further and defined. These themes were arranged into clusters and networks, and themes were combined and organized to develop global, organizing and basic themes. The themes, definitions and supporting quotes were examined by LEAP members to assess that the themes were representative of the data, and that the excerpts were aligned to theme definitions and descriptions. All thematic networks were defined and summarized and patterns in the data were interpreted. This process was undertaken separately for the patient data and the staff data. The thematic networks for the staff and patient data were then integrated, to form main themes from the data sets, which represented the most widely shared experiences of staff and patients as contributing to good QoL (including wellbeing, health and recovery) within the forensic mental health services (inpatients and community). This integration was undertaken with three members of the research team and the LEAP members. Integrated themes were refined, defined and reviewed to make sure that they accurately represented the patterns across the synthesized data. The synthesized thematic networks were developed arranged, defined and summarized. Staff and patient information weighed the same and the findings and recommendations made were based on this integration of data sets.

Strategies as recommended by Shenton (2004) were implemented to assess and address trustworthiness of the data, thereby examining the credibility, transferability, dependability, and confirmability of the data. Some of the strategies implemented were: the use and implementation of previously used procedures from other studies; extensive record keeping of analytical stages and use of detailed memos, to ensure findings were data driven and aligned to an inductive approach, and for transparency purposes; systematic checks by different members of the research team (including LEAP group) to assess that the findings were supported by the data and were representative of all the participants’ experiences; and use of independent researchers to verify the qualitative analysis undertaken and the conclusions drawn.

## Results

**Figure 1.**
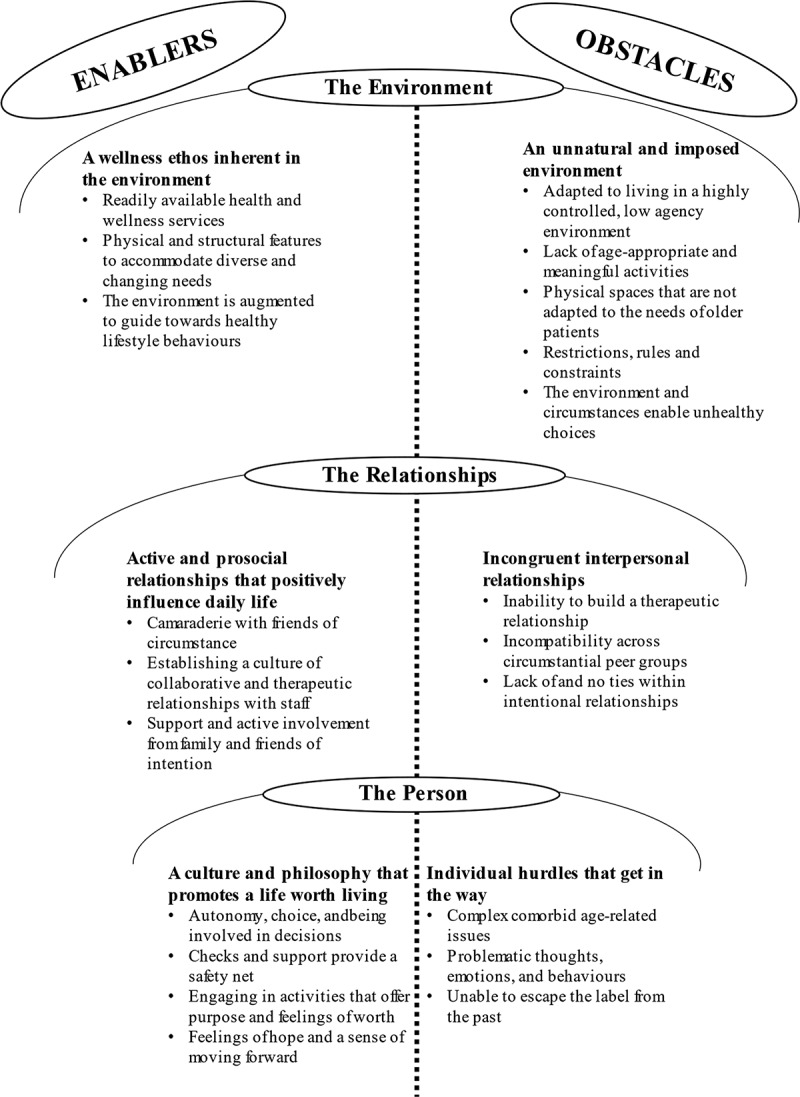
Themes and subthemes from integrated staff and patient data.

Figure 1 presents the themes and subthemes from integrated staff and patient data. As can be seen, there were two global themes: “Enablers” and “Obstacles”, which were further split into three organizing themes – “*The environment*”, “*The relationships*” and “*The person*”. These are the external and internal factors that are all interlinked and either act as facilitators/enhancers of, or barriers to QoL, health, and wellbeing. The enablers are therefore what need to be promoted and implemented, whereas the obstacles need to be diminished and if possible, removed from the patients’ lives. The findings are supported with quotes from the interviewees; those from patients are identified by the code P, followed by either LS for low secure, MS for medium secure, HS for high secure, or C for community. Staff are identified by the code S, and an abbreviation of their profession (as denoted in Table I) in the parenthesis.

### Enablers

Staff and patients identified the positive factors that promoted good QoL, health, wellbeing, recovery and facilitated progress, which were associated with either the environment, relationships, or the person.

### The environment

#### A wellness ethos inherent in the environment

This organizing theme is made up of three basic themes that represent resources that patients can access, to promote healthy choices.

#### Readily available health and wellness services

A range of different professionals were available: psychiatrists, nurses (mental and physical health), OTs, physiotherapists, dentists, opticians, dieticians, GPs, specialist nursing practitioners, and speech and language therapists.

S04(PMD):*You have got core trainees and psychiatry, psychiatrists, a GP coming in twice a week. We have got a dentist coming in and, and optician. There is a nurse you have got access to dieticians the diabetic nurse and tissue viability.*

This “readily available” provision was also seen with patients in the community where they are encouraged to have health checks and monitoring of their physical health. Support comes in the form of reminders as well as supporting and proactively organizing patients to get to health checks. As well as physical health care support there was also psychological and rehabilitation therapy with easy access for the patients. Such support was noted by both staff and patients as an accessible provision, so it appears to become a consistent and constant part of the service provided.

#### Physical and structural features to accommodate diverse and changing needs

Although this theme was evident in both the accounts from the patients and staff, it was more prominent in the narratives from the patients. Physical attributes of the built environment were important, for example, ensuite toilets, accessible showers or ramps, and not having stairs. Importance was placed on accommodating the needs of patients as they aged, e.g., as they became less able physically and mobility wise and needed extra support for daily activities.

S48(RMN):*I mean physical wise, we can bring in the physical accessories: crutches, wheelchairs, things like that. Lifts should they need bathing. Anything to do with getting out of bed, things like that. It’s just the physical stuff, mechanical things we can bring in.*

Having adequate space: communal, private, and outside space was required.

S07(OT):*Having plenty of space. They’ve obviously got their own bedroom and, multiple different sort of indoor and outdoor spaces.*

It was important to have an environment minimizing risk for patients from themselves and others; and that offers a feeling of safety for all.

P12(HS):*Bars on the windows, locked doors. I feel safer in here at the moment. I don’t think I’d do anything while I’m in here to myself or anybody else, but when I’m in here I feel safe. You’re watched all the time.*

The physical space also had positive psychological impact on the patients, who described that despite being a restricted environment, they still experienced positive feelings and a sense of ownership, where surroundings felt homely and not clinical.

S07(OT):*They want to kind of be here a lot of them it is kind of like a safe space, like a home for a lot of them … I think a lot of them are very content here, which, which, helps them feel sort of calm and safe.*

#### The environment is augmented to guide towards healthy lifestyle behaviours

Secure environments were organized in a way that embedded healthy lifestyle behavioural choices, making healthy opportunities easily accessible for patients. This removed barriers to accessing healthy choices that the general population might typically face, such as obtaining a gym membership, or deciding what food to buy, offering a path of least resistance and nudging patients towards healthy choices, e.g., lack of opportunity for smoking:

P22(MS):*I used to be a smoker, any smoker, so coming to an environment where I can’t smoke, has helped tremendously*;

promoting healthy eating:

P23(MS): *There have been the changes in the menu, I have to make good choices, the balance between the foods I enjoy as well as the healthy food;*

and an environment that supports physical activities:

S05(PSY):*Lots of focus on physical health, we’ve a really good physical health programme, we’ve big ground that people can walk in we’ve got the static physical health place, our OT run circuits*.

### The relationships Active and prosocial relationships that positively influence daily life

This organizing theme is made up of three basic themes that capture different interpersonal relationships spanning professional and peers/friends/family that are “*Enablers*”.

### Camaraderie with friends of circumstance

This theme was more widespread in the accounts of the patients and is about the supportive relationships with “friends” made through the circumstances they are in, such as other patients. They might not naturally choose these friendships, but they develop because their circumstances match up. There is a feeling of camaraderie and being part of the “group”, where supportive relationships with other patients contribute to QoL and wellbeing.

P20(HS): *I’ve got a lot of friends in here that support me. They talk to me, they play cards with me, play other games. They’re always asking me if I’m all right. If I’m looking down, they come and talk to me or make me a brew. They’re always asking if I’m all right.*

The participants discussed the importance of access to other friends of circumstances such as volunteers (e.g., befrienders, Samaritans) or chaplaincy who become “friends” and part of a support mechanism.

S06(N/CLIN):*Those who don’t have any family members, they have the Samaritans who come in and they will sit with them. One of the things that works well for me [a Chaplain] is being a friend to the service user. Just being a friend.*

### Establishing a culture of collaborative and therapeutic relationships with staff

QoL, health, wellbeing and progress was enhanced by the characteristics, attitudes, and actions of staff, who created a supportive environment. This included staff being caring, empathetic, compassionate, and putting patients first. The patients considered staff people to trust and confide in. Patients could talk to staff about problems, seek advice from them, socialize with them, or talk about shared interests.

P16(HS): *Well, there’s a couple of staff nurses I like talking to. There’s a few of them in here I can talk to because they’re right down to earth with you…they’re like you and they have a laugh*.

Staff get to know patients well and understand them and their experiences. It seems favourable when the staff are of similar ages. Some patients are in hospital for such a long time, that the staff become like “family”; this closeness contributes to developing a successful therapeutic relationship.

S17(RMN): *One chap he’s just gone over 55, I’ve nursed him for years, and I think for him because he hasn’t got a lot of family, it’s actually building relationships with the staff, they’ve become his surrogate family, and having those positive relationships has been good for him.*

#### Support and Active Involvement from Family and Friends of Intention

Family and friends external to inpatient settings represent patients’ intentional relationships. These chosen and mutually agreed friendships support the patient through visits, maintaining a presence in their lives, and helping with care planning.

S03(PMD):*Family contact … especially if family has been important for them, and has been supportive, if they got an extended family, children, grandchildren, maintaining that earlier on … It’s ensuring that they have got good family contact, good family support, maximizing their quality of life is more likely to get them to progress.*

This contact across different family members was identified by the patients as something really important, including active involvement from children:

P01(C):*Yes, the relationship I’ve got with my kids. Yes, that’s essential for me;*

grandchildren:

P31(C): *Seeing my grandkids and children, that’s my best thing. Just seeing my grandkids and children*;

parents:

P10(C): *It’s nice to meet my mum, my dad … . They give me good, in terms of well-being, knowing they’re safe and happy, and keep me positive*;

and siblings:

P22(MS):*Yes. I have contact with one of my sisters and her son, fortunately enough … she’s quite happy to help me out and support me*.

Friends of intention external to the friends of circumstance, were also identified as an important support mechanism for the inpatients and was also seen out in the community. Here, they could provide a social mechanism to support the patients and help them move on.

S15(C/RMN): *Meeting with friends, for a coffee or things like that, simple things are quite important for some people … you know I think it’s important for them to have friends to support them it helps their wellbeing and helps them move on with encouragement.*

## The person

### A culture and philosophy that promotes a life worth living

This organizing theme includes four basic themes. These factors are at an individual level; underpinning this is a culture and philosophy that enable patients to feel valued, ensuring their lives have worth and meaning.

### Autonomy, choice, and being involved in decisions

Contributing to QoL was patients being able and empowered to make choices and decisions about their lives. The culture is one of enabling individuals to be active participants in their lives, care and treatment, and not simply being passive and having everything imposed on them.

S06(N/CLIN):*They sort of empower them, when they have morning meetings, they allow the older ones to chair the meeting, give them some form of responsibility, and accountability.*

Staff talked about how, for the older patients, the philosophy was to use the Good Lives Model (Laws & Ward, 2011) as a framework for their care, placing emphasis on agency, i.e., autonomy.

S05(PSY):*The patient matters in this, so being really clear with the patient about what they want, what they find value in, so I think the model I that I drive particularly for all of my patients, but I find works very well for the older group, is the “Good Lives” model.*

Patients talked extensively about autonomy and the importance of having freedom to decide what they want to do and being able to do this.

P12(HS):*Do models. Go for a walk outside. Doing things, I enjoy. Not being pushed into things, having a bit of choice of what I do. Being able to do what I want when I want [gives good QoL].*

## Checks and support provide a safety net

This theme was noted more by patients than staff and mostly related to patients in the community, who highlighted the value of having support in place to act as a safety-net or back-up, above and beyond the day-to-day care that they have access to. This safety-net gave patients a sense that support is there to draw on if needed, giving them confidence that someone “has their back”.

P33(C): *Having the back up of my team … because if I’ve got something to fall back on, they’re always there. It helps me function better knowing that if I feel that there’s something that I’m not comfortable with, for whatever reason, I can chat with them about it. It’s like having a safety net.*

Back-up can also occur in the form of checks, such as on medication, physical health, activities and mental health. Having staff in the background allows them to monitor patients, identifying potential problems early, and offering support mechanisms if required.

P01(C):*It’s usually just like dieting, make sure—I don’t eat too much takeaways. Physical health. Walk a bit more. Check the mental health to see if the mental health’s all right.*

## Engaging in activities that offer purpose and feelings of worth

Roles and responsibility facilitated feelings of being valued, respected, and doing something worthwhile. This enabled a real sense of purpose, worth and a reason for being, where activities contributed to patients’ sense of agency and identity. Bookbinding gave P12 a sense of status and ownership where he takes on the social role of expert in a given skill, and with this gets a sense of worth and being needed by others.

P12(HS):*I really enjoy the graphics, bookbinding. I was the one they’d come to if they wanted anything doing in the workshop. It would be staff, patients, sending books down to be stripped, taken all apart, resewn or new covers. I used to do all that. I really enjoyed it. I’m useful, proud.*

Activities important specifically to the patient, and congruent with things of importance across their lives were particularly valued. This brought meaning to activities and gave a sense of autonomy and choice through exploration of their own identities instead of being pushed to do things that did not fulfil their sense of self.

S07(OT):*Doing meaningful activities that, that they enjoy. We have got people who, are musicians, who are in their 70s and it’s trying to encourage keeping that identity going.*

Activities were useful in keeping patients’ minds occupied and through focusing on the activities, they are then not thinking about other problems that they may have.

P18(LS):*I like drama, sort of plays and that. Poetry. Stuff like that. Takes my mind off other things. Other things for a little while. Takes my mind to other things.*

## Feelings of hope and a sense of moving forward

A sense of hope for the future, and a positive future focus enables patients to feel a sense of worth and something to strive towards.

S24(SW): *We try as best to create a recovery-focused environment. You know, those things like hope, hope for the future, purposefulness, meaning, things around identity.*

In the narratives there is a real sense of patients having goals and a future to aim for. It was important for patients to have positive states of mind, and several talked about how: *Positive frame of mind* [P04(C)]; *Positive thinking* [P08(LS)]; *Positive mindset* [P06(LS)]; and *Positive attitude* [P10(C)] was associated with good QoL, wellbeing and progress. There is a feeling that the underpinning philosophy must be a future-focused one, with a mindset of having things to look forward to, as opposed to dwelling on the past and on things that cannot be changed.

P04(C):*The main thing is optimism and a positive state of mind. I’m getting older now, I sometimes think about, the end of my life, I try not to do that as much as possible. There is always something new to keep me going and make me look forward rather than backward.*

## Obstacles

The staff and patients all identified factors that prevented or obstructed good QoL, health, wellbeing, recovery and progress, associated with the environment, relationships, or the person.

### The environment

#### An unnatural and imposed environment

Safety, rehabilitation and criminal justice requirements create an unnatural and imposed environment, so choices are dictated and defined by staff, resulting in a highly controlled, and low patient agency environment.

##### Adapted to living in a highly controlled, low agency environment

Participants described that over time they adapt to living in this controlled environment. This involves being institutionalized, which creates a dependency on the system and other people and is particularly problematic for those who have a long length of stay.

P05(LS):*It’s a sad thing to say, but the regime of the place. To get institutionalized is wrong, but I’ve been, this section I’ve been in 15 years, I’m institutionalized.*

This sense of institutionalization followed patients from inpatient settings to the community, where the feeling didn’t simply disappear when physical restrictions were removed.

S01(C/RMN):*Some people, even in the community, still think in a very institutionalized way, they still see themselves as part of the system, as being controlled, monitored.*

Patients adapted to having structure and being told what to do, developing a reliance on others, potentially associated with a loss of skills. The system and structure designed to support patients to manage their mental health conditions can leave them ill-equipped to move on and progress.

P07(LS):*Living in a nice secure, like this one and an en suite and quality of life, food on the table, no bills to pay. I don’t want to move from here, it scares me that does, it scares me moving from here, because I might put the cooker on and forget to turn it off.*

For long-term patients, the world beyond their inpatient setting has changed, which is frightening for some. Unknown developments in the community such as technological advancements (computers, phones and the internet) can create fear and worry, and some patients may prefer to stay as an inpatient where they feel safe.

S48(RMN):*Being scared of going out and facing a big, wild world, technology is changing that fast, maybe can’t deal with it. Things like phones, using computers, using iPads, anything at all to do with televisions, it’s just they don’t know. Even just going outside, how fast and hectic it is.*

##### Lack of age-appropriate and meaningful activities

This unnatural and imposed environment affords a lack of appropriate activities for patients, particularly meaningful ones. The restricted environment takes away individual choice. The lack of available things to do, or restrictions (e.g., like simply not being able to go outside) leads to feelings of boredom and monotony.

P03(MS):*I’m a bit bored twiddling my thumbs and not enough activities. I really need to feel like I’ve got a sense of freedom to get outside and be in an open space. Really missing like the walks and going outside for a long walk, that’s what I’m really missing.*

Certain activities like arts and crafts were not suitable or age-appropriate: “*Some [activities] are for five or six-year-olds. It’s glitter everywhere. Painting. Making cards*” P12(HS). Physical issues associated with ageing could also render some physical activities unsuitable for older patients.

S08(PSY): *You know, the, the young lads that are awesome footballers and basketballers they can do that all, and it is set up great for them, but for people that want to do, lower-level introduction to sport or just gentler, more gentle things, there isn’t as much access.*

##### Physical spaces that are not adapted to the needs of older patients

Some physical environments were not suited to patients’ needs, and patients don’t have the agency to adapt their environment. The environment cannot accommodate physical issues associated with ageing (frailty, poor mobility) or declining physical health. Participants described not having handrails, walking sticks, Zimmer frames (hand-held frames to aid walking) hoists, footstools, raised toilet seats/chairs, and that furniture was fixed and not moveable.

P18(LS):*I like getting in the bath, out the bath but it’s standing up after I’ve sat down. I cannot stand up again. I have to be lifted up. I can’t reach. Because of everything I very rarely get to have a bath. I’d like to have to an invalid bath. A hoist, a sort of hoist.*

Buildings were described as dated, multilevel, having too many stairs, having poor access to outside, and not being able to accommodate wheelchairs in the community:

S01(C/RMN):*His mobility is poorly, he has arthritis, and glaucoma so his eyesight is poor*, *he lives on the 2nd floor, and it’s not ideal for him, going up two flights of stairs;*

and on the wards:

S04(PMD):*If they have got physical health problems, not having en suite bedroom may get in the way of their quality of life. Our rehabilitation units are all upstairs. Individuals, who had problems with knees they need a lift, rather than going up and down the stairs 10 times a day.*

##### Restrictions, rules and constraints

Participants expressed that the system, processes, resources, or lack of provision, can be a barrier. Restrictions and rules preventing patients from accessing things that most take for granted, such as technology, visiting and seeing family, deciding what to eat, going out, and being able to do activities they want.

S07(OT):*It is the restrictions of secure care, so they can’t go out and visit family, don’t have full control of what they eat whether mealtime or snacks, lack of opportunities to go out to do things.*

The restrictions and rules reduced patients’ freedom which they felt impacted their QoL, wellbeing and progress negatively.

P03(MS):*For me to improve my quality of life even more would be to be able to go outside…. Having more freedom, more opportunity to go outside. Lack of freedom, I feel a bit cooped up.*

Patients experienced additional restrictions due to COVID-19. Activities were reduced or stopped completely, as were visitors, leave and mixing with other patients. For some in the community, they found themselves back to a restricted environment, almost one akin to being in the unit. The restrictions also prevented some transitions to the community’.

P07(LS):*If it wasn’t for the COVID, I’d be moving on now [to community], because of COVID I think they’ve locked down until about January now.*

Policy, processes, administration, and paperwork also impeded patients. This was related to risk and risk assessments, which was a barrier for patients restricting them further.

*S03(PMD): Special permission, that can be time consuming, sometimes people think why bother, people say, “I’m not going to fill out three forms, to justify myself, to get this person one hour of leave, I won’t bother doing it, rather tell the patient it can’t be done, policy won’t allow for it.*

##### The environment and circumstances enable unhealthy choices

The environment facilitated poor lifestyle choices, e.g., access to take away food, the on-site “tuck” shop (selling confectionery or snacks), lack of exercise facilities, and provision of meals that were not always healthy.

P05(LS): *Food-wise putting on weight, I’ve put on weight. I don’t want to become obese. A lot of the problem is obesity, when they make things easy to do. I’ve got loads of bad eating habits.*

The restrictive nature of the settings makes choices such as takeaways highly valued and rewarding as they represent a treat and a way of exercising some choice or control, in comparison to someone not within a unit, who has wider range of options, choices, and treats in more aspects of their lives.

S12(PHY): *A lot of our patients really struggle with obesity, but they’re still able to order takeaways, because there isn’t that want to come and restrict them even further and take a little bit of choice away from them.*

The restricted environment creates a situation where patients have a disposable income, and little options to spend it. Patients then buy large quantities of unhealthy food from the tuck shops. S45(I/RMN): They go to the shop and come back with a trolley load of fizzy drinks, crisps, biscuits and stuff, because they have money. They’ve got money, and you could see the obesity.

#### Incongruent interpersonal relationships

This organizing theme is made up of three basic themes which represent an absence of, or poor, pro-social relationships with three different groups: staff, fellow patients, and family and friends.

##### Inability to build a therapeutic relationship

An absence of therapeutic alliances, and not agreeing on care planning impeded patients’ QoL, health, wellbeing, and progress. Some patients were unable to create the therapeutic relationship because they did not get on with the staff, *“I’ll just say there’s a couple of staff who I don’t really get on with”* P16(HS). A lack of shared goals and understanding of their care was also problematic resulting in patients feeling excluded from their own care. On occasion patients simply felt they were not listened to, rupturing therapeutic relationships. Patients were “done to”, i.e., staff make all the decisions, with no collaboration and as “experts”, staff pull rank on decision making and what happens to the patients. Patients become passive recipients, are not given a voice and lose their sense of agency.

S12(PHY): ‘*The doctors go “well we will try it again it will be fine”, and kind of overrule them. I don’t think their voice is always heard*. *It takes away that autonomy and that drive in them, it’s like what’s the point of me having a voice if everyone else is going to make that decision for me.*

High staff turnover, use of bank staff, and lack of staffing time due to low staffing numbers or demands of a busy ward prevented opportunities to develop staff-patient relationships.

S40(OT): *Having time for people is really important, so I know we’re all busy, but I do think people need a bit more time…it comes down to time and resources, and everyone is running around being busy and, so, I guess a bit more time and maybe a bit more resource would help.*

Older patients found it difficult developing therapeutic relationships with staff who were a lot younger than them. They felt that they couldn’t relate to younger staff, which suggests having staff of a similar age could allow for more shared experiences and understanding with them.

P16(HS): *The staff that should be on that ward shouldn’t be kids … It just needs more staff of the same ages but older generation because you understand each other.*

##### Incompatibility across circumstantial peer groups

Patients described not getting on with everyone or experiencing the wrong mix of people, such as younger and older patients who did not integrate well together. This could be due to lack of common ground or shared experience or a feeling of being the minority and not fitting in with the group.

S04(PMD): *Peer groups, some of the older gentlemen will say “that unit is full of young people, who all talk a certain way and are interested in certain things, and I don’t feel like I fit in”. So, they may not feel part of the, the group as a whole … they are in a minority.*

The environment becomes unnatural as patients find themselves amongst people, they wouldn’t necessarily choose to be with but have to be. Furthermore, the social dynamics and positioning of older patients as a minority could develop into bullying. Coupled with an inability to remove themselves from such situations this could lead to maladaptive responses such as violence.

P34(HS): *People bullying me, trying to intimidate me, I’ll take so much. In the past, I’ve just swung at people, hot water, that was my way of dealing with things.*

Inpatient settings can exist in a state of flux as patients are admitted in and transferred out; the changing dynamics often involve new arrivals who are very unwell or violent. This could be intimidating for older patients, who may avoid social spaces to keep out of trouble, and then risk isolating themselves, and reduce the chance of positive social interactions.

S35(SW): *You have new people coming in who are very acutely unwell or creating disturbances, that’s difficult for the older guys sometimes. The older ones seemed to be more settled, so that’s something. But they tend to get a bit frightened, hide away in their room, become isolated.*

#### Lack of and no ties within intentional relationships

Some patients experienced an absence of any intentional relationships, due to estrangement from families, relationship breakdown, disconnections or through being shunned or rejected.

P07(LS): *No, my three children don’t want to know me, they’re all grown up now and they’re only after one thing, the money side.*

The long length of time patients are in units can break social connectedness with family and friends; many patients missed the opportunity to build a family life of their own. They become isolated from family groups and social settings.

S36(PSY): *Some of them seem to have less contact with family. I think a lot of our older adults have been in the system for a long, long time, so either haven’t had opportunity to start their own family. Or those ties have been lost over the years. I think we see an awful lot more isolation with this group outside, in terms of outside connections.*

An issue associated with patients’ ages was that family members had passed away, removing a valuable source of external support in the community that could help them progress.

P21(HS): *I used to have my aunty, and my uncle, and my dad, and my mum, I don’t anymore, they’ve passed away. So, I wouldn’t be able to cope outside myself.*

Friendships of intention were difficult to maintain as patients were in secure units for a long time. For some patients the curtailment of these relationships was an active choice.

P15(LS): *I don’t have any—my friends I’ve put aside. I’ve put my friends aside … I’ve got no friends that I could really consider to be worthwhile outside in my local area.*

#### Individual hurdles that get in the way

This final organizing theme is made up of three basic themes, which are about patients’ individual characteristics and “the self” that can impact negatively on their QoL, health, wellbeing, and prevent progress.

##### Complex comorbid age-related issues

Co-morbid age-related issues experienced by patients were widespread, particularly physical issues associated with ageing, such as heart and respiratory illness, arthritis, chronic illness, and serious diseases.

S10(I/RMN): *They’re going to have more complex physical health issues, so a lot of our gentlemen have got diabetes, cholesterol, blood pressure, heart conditions, COPD.*

A key concern is age-related cognitive changes, due to organic decline and onset of diseases such as dementia, Alzheimer’s, Parkinson’s, and other neuro-degenerative diseases, general decline in cognitive ability, slower cognitive processing as patients age, or simply a poor memory.

S04(PMD): *With cognitive problems or with the diagnosis of dementia, will progressively get worst due to the nature of the illness, with a formal diagnosis of dementia, progress might be a misnomer, you know, just by the virtue of the illness, you know they will deteriorate over time.*

It was also apparent that some patients experienced age-related general physical deterioration such as frailty, falls, poor eyesight, loss of hearing, and lack of or poor mobility.

P01(C): *They need to look at my mobility and my eyesight. Both of them are degenerating.*

##### Problematic thoughts, emotions, and behaviours

Participants talked about numerous adverse emotions that they felt contributed to poor QoL, health and wellbeing, and that could hamper progress. Examples of this include guilt:

S01(C/RMN): *The over 55 year old gentleman I’d worked with he had so much guilt over the things he had done in the past*;

negativity and anxiety:

P05(LS): *I haven’t got it (quality of life) here. I’ve got negativity and depression and anxiety and panic*;

hopelessness:

P03(MS): *Barriers to quality of life… feeling hopeless… I feel like giving up and being self-destructive;*

shame:

S01(CRM): *They have this massive sense of shame about themselves, so that prevents them from moving on;*

and low self-esteem:

P04(C): *I’ve not got very good self-esteem, that overwhelms me, and I just feel I’m good for nothing and life isn’t worth living*.

There was also an element of problematic behaviours associated with self-sabotaging. This could result in relapse, violent and abusive behaviours, or for those in the community, taking drugs. For some, self-sabotaging behaviours were a way of remaining in or going back to the secure unit where they felt they wanted to be, but that ultimately prevented progress.

S10(RMN): *Like that revolving door so someone that keeps coming back into services or they deliberately avoiding compliance with medication to become unwell so they can come into a service where they’re familiar with … They feel like they look at nursing staff like a family they don’t want to leave here and they will try to self-sabotage.*

##### Unable to escape the label from the past

This theme was predominantly found in the staff data. The stigma and labels of past behaviours, offending, mental health, and patients’ age could become particularly stuck to older patients, even though for many the offending and behaviours were many years ago and they had completed a considerable amount of therapeutic work since.

S27(C/RMN): *Stigma reduced is quite a large part of that … that’s a massive barrier when people have, been in hospital for a large amount of time and they come out. People who are older and maybe committed their crime when they were 20 that one incident, maybe, 40 years ago would still be held and they would still not be able to access things.*

It was problematic for patients when they were stigmatized with a “*mental health*” and a “*forensic*” label. Decisions were made based on these labels.

S29(C/SW): *We’re addressing the stigma of mental health, but the stigma of forensic mental health is another issue altogether. Clearly, forensic puts the frights into people. I wouldn’t like to have to say I’m a forensic mental health patient really. Maybe we ought to get rid of that word “forensic”.*

### Discussion

To date there has been little research that has examined QoL, in terms of health, wellbeing and recovery for older forensic mental health patients, and what is required to promote these and enable individuals to progress. This is important to do, given the increase in the numbers of older forensic mental health patients, and because their needs are diverse and complex, straddling “*forensic*” “*mental health*” and “*older people care*” service provision. Improvements in QoL for forensic mental health patients can augment perceptions of recovery that in turn promotes scope for progress (Barsky & West, 2007). With a shift towards recovery-focused practice in forensic mental health settings, QoL and wellbeing are identified as important outcomes (Mann et al., 2014), and psychiatric and medical care intervention aims to optimize QoL as a treatment target (Schel et al., 2015). QoL is deemed to be a positive protective factor, linked to risk reduction as it is associated with reducing both short- and long-term recidivism (O’Flynn et al., 2018). However, it has also been argued that QoL should be seen as a broader concept that considers more rehabilitative and humanitarian aspects of treatment (Vorstenbosch & Castelletti, 2020). The current study generates valuable knowledge and findings around the environmental, relational and individual factors, arguably some of which could be valid for the whole group of forensic psychiatric patients regardless of age (e.g., relationships, individual autonomy), with other findings unique to older patients (e.g., environmental needs of the elderly), that are important to promote and maintain QoL. This was based on the perspectives of the patients themselves as well as the staff who work with them. Here taking a critical realism lens, the causal mechanisms (environmental, relational and individual) that have the potential to drive social events, activities and phenomena are suggested and theoretical accounts are offered for the events and effects that have been observed and experienced.

One issue highlighted was the imposed environment the patients find themselves in. This can foster a sense of low agency, with restrictions and levels of security deemed as detrimental in terms of QoL, linking to the findings of previous studies (Long et al., 2008; O’Flynn et al., 2018). This theoretical perspective points to causal mechanisms that go beyond individual choice and that shape agency in a particular way. As such it is important to situate older patients within the context of environmental restrictions and rules. QoL can be improved in forensic mental health populations through increased autonomy, privacy, personal control, and when patients are in charge of their activities of daily living (O’Flynn et al., 2018). Patients’ autonomy needs to be encouraged, although this can be challenging in secure environments where there is tension between security, safety, and the provision of a therapeutic environment. Services need to be therapeutic and secure, providing a positive and supportive environment, where clinical care and therapy can be safely delivered (Seppänen et al., 2018).

Structurally, the environment needs to be suitable for older patients in terms of design (e.g., wheelchair access, stairs, ensuite bathroom) and through the provision of equipment (e.g., handrails, moveable beds, seating) to accommodate for deteriorating physical health, reduced mobility, frailty, and decline in hearing, sight, and cognition. These were the contextual conditions for particular causal mechanism to take effect in relation to quality of life, wellbeing, recovery, and progress. The environment also needed to offer safety and reduced risk. The CHIME-S framework (Senneseth et al., 2022), that guides recovery-oriented practices in forensic mental health services, identified how service users needed to feel safe and secure, including protection from hostile people and environments. The authors also noted the relevance of the active practice of self-management of risk within this. In the current study individuals commented feeling safe in their environment by being watched reducing the risk of them doing something harmful, and reducing the risk posed by others, who were also being watched. Shepherd et al. (2016) based on a review of the literature found that a personal sense of safety was a prerequisite for any recovery process, and that this could be provided by the individuals physical environment, highlighting the importance of this concept within forensic mental health environments.

The environment also needs to offer a sense of comfort, be homely (i.e., “home-like” surroundings that are comfortable and welcoming) for patients, with private space, but also good communal and outdoor areas to foster social interactions. Privacy and access to adequate personal space have been identified as important aspects of the physical environment for forensic mental health patients (Olausson et al., 2021; To et al., 2015). Patients dislike a clinical and sterile environment, as it doesn’t provide comfortable or homely surroundings (Olsson et al., 2015; Völlm et al., 2018).

Embedding health and wellbeing promotion so that patients avoid and reduce engagement with unhealthy lifestyle choices such as smoking, inactivity, over-eating, and poor diet (unhealthy food choice, snacking, take away food) is important. From a critical realist viewpoint, generative mechanisms are embedded within social structures and are contextually contingent, and work through people’s actions; social structures provide the conditions that constrain or facilitate health-related activities (Angus, 2012). There is the issue that the restrictive nature of the settings makes choices such as takeaways highly valued, yet there is evidence of an association between takeaway food and obesity in secure settings (Oakley et al., 2013). It has been proposed that restrictions could be put in place as a way of addressing this, although, human rights and autonomy issues arise from restricting choice (Kasmi, 2009). Perhaps including patients in discussions concerning food policies on wards may address the ethical dilemmas that are observed when imposing limitations, such as limiting access to takeaways (Oakley et al., 2013).

For older adults, easy access to gyms and exercise classes is beneficial, particularly where the exercises are adapted and tailored for their needs. Many units now implement a non-smoking policy so that smoking is only allowed on community leave, and this has been found to reduce smoking (Pedersen et al., 2020). Healthy eating also needs to be embedded within the service provision. Generally, meals provided are high in carbohydrates and fat, with few vegetables; portion control is problematic (large), and patients frequently take advantage of being able to order take away meals and additional snacks on top of their daily meals, resulting in weight gain and obesity issues (Huthwaite et al., 2017; Long et al., 2009). However, there remains a challenge of enabling healthy lifestyles in the community, where exercise facilities are less accessible and support to access is limited (Pedersen et al., 2020).

Within service provision, recovery models of care look to incorporate a holistic approach to recovery that embeds and values social relationships and connectedness (Jacob, 2015). Relationships were important for the patients—when they were positive, fulfilling, and contact was regular they aided QoL. Therapeutic relationships with staff were key for patients, something commonly identified to enhance recovery (Marshall & Adams, 2018). One of the six key recovery process that define the CHIME-S framework related to connectedness, and patients being a part of the community on the ward, and importantly their relationship with staff and the quality of this (Senneseth et al., 2022). Research suggests that therapeutic relationships in forensic settings can be poor and that this can impede recovery (Markham, 2021). In the current research, staff/patient relationships identified as problematic impacted negatively on patients. This was observed by Senneseth et al. (2022), where service users identified feelings of “them and us” and poor relationships were highlighted as a barrier to recovery; given that a positive association between therapeutic relationships and QoL for forensic mental health patients exists in the literature (O’Flynn et al., 2018), the importance of optimizing therapeutic relationships is clear. The social climate of a ward has been identified as promoting positive wellbeing and treatment outcomes, and one the dimensions associated with social climate is “*Therapeutic Hold*” (Schalast et al., 2008), which the quality of therapeutic relationships comprises part of. Strong therapeutic relationships are predictive of engagement, positive treatment outcomes, service user satisfaction, and may reduce symptomology and increase likelihood of medication adherence (Bressington et al., 2011; Horvath, 2000; O’Flynn et al., 2018).

Other relationships also viewed as beneficial to the patients were those with peers—or friends of circumstances—as well as with family and friends (intentional friends). Peer support and patient cohesion was observed as important and necessary, and this could be linked to social climate, and the dimension “*Cohesion and mutual Support*” (Schalast et al., 2008), which relates to the extent that others offer positive support and are cohesive in each other’s rehabilitation and recovery (Puzzo et al., 2019). Positive relationships and attachment to supportive individuals was also observed outside of the hospital and identified as an important element of the recovery process (Mezey et al., 2010; Nijdam-Jones et al., 2015). Relationships with family and friends provide opportunity for patients to connect with their evolving sense of self, and with the outside world and promote positive recovery outcomes (Gillespie et al., 2021).

On an individual level, autonomy, choice and being involved in their care and treatment decisions promoted QoL, health and wellbeing for the older forensic patients. In many ways compulsory detention conflicts with the concept of patient autonomy and likewise intrusive monitoring in the community can restrict autonomy and agency (Carroll et al., 2004). Although detention and restriction are needed for reasons of safety and risk, patients should be empowered, so that they are involved in decision making and are able to make choices. Contemporary models of recovery for forensic mental health patients have the principles of autonomy, empowerment and self-determination embedded, and are seen as essential for achieving positive outcomes. One such model is the Good Lives Model (GLM) (Ward & Brown, 2004; Ward, 2002), which doesn’t solely focus on risk, but places emphasis on human agency, autonomy, abilities and strengths. Emphasis is on the conception of a “good life” and “client individuality” where patients’ values and preferences are realized and taken into account (Barnao & Ward, 2015).

The participants also discussed the importance of meaningful activities. They kept patients occupied, prevented boredom, and helped remove negative thoughts. Previous research has shown a positive association between engagement in meaningful activities and QoL for forensic mental health service users, particularly in relation to psychological health and social relationships (O’Flynn et al., 2018). One of the recovery processes defined in the CHIME-S (Senneseth et al., 2022), was meaning in life, which included meaningful use of time on the ward. They suggested that recreational activities, paid work, leisure activities and spending the day in a purposeful way enhanced quality of life and supported mental health. It has also been suggested that engagement in practical and meaningful activities enables the individual to live independently and have a sense of purpose and identity (Roberts et al., 2015). Within the narratives of the patients and staff, this sense of purpose from activities was clear, giving patients a reason for being, where activities contributed to their positive sense of agency and identity.

An obstacle identified was that forensic mental health patients find it difficult to escape labels of forensic (offenders), mental illness, and old age, and the accompanying stigmatization which can prevent recovery, hamper discharge, and persist in the community (Mezey et al., 2010). Senneseth et al. (2022) identified there were some barriers to personal recovery, one being negative identity experience and stigma. It was argued that stigma could be a barrier to contact with family and friends and can have a negative effect on self-esteem. Strategies need to be put into place to enable patients to overcome any negative identity of stigma that they may experience. Patients need to be viewed as capable of change, and not defined by their crime, illness, or age, and forensic service provision must take a non-judgemental and non-stigmatizing approach to treatment (Marshall & Adams, 2018).

The findings of this study need to be considered within the context of its limitations. The interviews were conducted with individuals from eight NHS trusts in England and therefore does not include other types of forensic service providers (e.g., independent). A strength of the study was the large sample size gaining representation from patients in different security levels and staff from relevant professions. By taking a qualitative approach this provided a rich dataset and allowed for a systematic unpicking of all these individuals’ experiences and the co-construction of knowledge, however, as it was based on a selection of accounts in specific contexts it may not be generalizable.

This methodological approach enabled an exploration and understanding of a phenomenon from the perspective of those experiencing it, but it does not offer the ability to make direct cause and effect inferences in relation to the specific outcomes of interest such as QoL, wellbeing, recovery and progress. The methodology was suited and appropriate for addressing the aims and research question posed. Key concepts were operationalized in interviews via the structured questions posed, but this may limit emergent themes compared to other approaches such as grounded theory. The patient participants, for reason of risk, sometimes had to be interviewed with staff members present (this was explained as part of the recruitment and consenting process, with patients given the option not to participate if they did not agree to this), which could have influenced some of their answers. Patients may have therefore felt they could not discuss negative factors as this might influence their ongoing care potentially leading them to focus on the positive aspects more. Participants also took part of their own free will, and this may introduce a selection bias. Finally, it was not possible to have access to the details regarding the length of time that patients stayed within the secure units. Length of stay could be a confounding factor for subjective quality of life and as such the lack of this data limits the discussion related to the interpretation of results around this subjective factor.

### Conclusions

This research applied qualitative methods to obtain in-depth narratives and views from staff and older forensic mental health patients, who, given the difficulties and complexity in trying to engage and include them in such projects, are commonly not given a voice through research. The environment, interpersonal relationships, and individual characteristics of the patients need careful consideration in relation to service provision and care, given their interconnectedness and impact on patients’ QoL, health, and wellbeing. A “wellness” environment is required, whereby physical and mental health is assessed and addressed (particularly issues and needs associated with the ageing process). The environment needs to be adapted to cater for changing needs for people as they get older and be such that it promotes healthy lifestyle behaviours (e.g., good diet, exercise, no smoking). In addition, prosocial relationships need to be a feature in patients’ lives; this includes with family and friends, peers on their units as well as therapeutic relationships with staff and professionals. On an individual level, there needs to be the opportunity for autonomy and choice, where individuals have access to meaningful activities and so have a purpose, where a sense of worth and a feeling of hope is enabled and supported. This aligns with the key recovery processes in the CHIME-S framework (Senneseth et al., 2022) and reflects the personal recovery processes required for older forensic mental health patients. Overall, provision is required of the least restrictive, but safest environment that can cater for forensic, mental health, and older persons’ needs, where social relationships and therapeutic alliances can be fostered and developed. Then, by empowering individuals and promoting a sense of autonomy, older forensic mental health patients can experience personal recovery, positive QoL and progress successfully.
